# A superior articular process morphology of 5th lumbar vertebra prone to screws placement failure: an anatomical study of 299 patients

**DOI:** 10.1186/s13018-022-03403-y

**Published:** 2022-11-24

**Authors:** Xiang-Ge Liu, Pei-Jie Liang, Han-Hui Liu, Guang-Fu Chen, Xiao-Dong Zhao

**Affiliations:** Department of Spinal Surgery, Fosun Group, Foshan Fosun Chancheng Hospital, Foshan, 528000 China

**Keywords:** Superior articular process, Morphology, 5th lumbar vertebra, Pedicle screw placement failure

## Abstract

**Purposes:**

This study aimed to investigate whether the morphology of the superior articular processes of L5 vertebra affected the accuracy of pedicle screw placement by reviewing 299 patients who had undergone L5 pedicle screw fixation over the past 12 months and measuring relevant parameters.

**Methods:**

We retrospectively analyzed patients who underwent L5 vertebra fixation at our spine surgery department from October 20, 2020 to October 20, 2021. Patients with spondylolisthesis, spondylolysis, and scoliosis were excluded. Parameters associated with the superior articular process were analyzed, including Mammillary process-Spinal canal Distance (MCD), Inter-Facet Distance (IFD), Inter-Pedicle Distance (IPD), Zygapophysial Joints Angle (ZJA), Superior Articular Width, and Lateral Recess Transverse Diameter. The L5 vertebral body was reconstructed by Mimics 21.0, and the simulated L5 screws were inserted at multiple entry points to measure the Maximum Safe Transverse Angle (STA^max^).

**Results:**

A total of 299 patients who underwent L5 vertebra fixation with 556 pedicle screws were analyzed. An MCD < 6 mm was associated with a significant increase in screw placement failure rate and decrease in ZJA. The MCD was positively correlated with IFD. No significant change in IPD was observed. Mimics software analysis showed that the STA^max^ decreased with a decrease of MCD. When WBV < 6 mm, 93% of the trans-mammillary vertical line was located within 50% of the pedicle.

**Conclusions:**

The superior articular process tended to narrow the spinal canal and exhibit a steep and a “cloverleaf” morphology when the MCD was < 6 mm. This morphology increased the risk of operator mis-judgement resulting in screw placement failure. Assessment of the relationship between the trans-mammillary vertical line and the pedicle represents a simple method to predict abnormal morphology of the superior articular process before surgery.

## Introduction

Pedicle screw fixation is essential for maintaining spinal stability in spinal surgery. Breach or perforation of the pedicle cortex is a widely acknowledged complication of screw placement, with an incidence rate between 6.5 and 13% [1–4]^.^ Perforation of the medial and inferior wall of the pedicle cortex is more likely to lead to nerve stimulation, leading to postoperative nerve pain [5]. Most patients with postoperative pain due to screw placement failure need reoperation. Even if pedicle perforation does not result in neurological symptoms, pedicle wall damage reduces the biomechanical strength of pedicle screw fixation and accelerates the progression of degeneration of adjacent segments of the lumbar discs [6–8].

Common entry reference markers for pedicle screw placement include the mammillary process, accessory process, and lateral margin of zygapophysial joints. The commonly used methods of pin entry points include the Roy-Camille [9], Magerl [10], Weinstein [11], and “^”-shaped crest techniques. The “^”-shaped crest put forward by Professor Xin-ru Du is a crest structure where the pars interarticularis converges at the accessory process, which appears almost constantly in L1–L4, but only in 81% of L5 vertebra.

It is well-established that the accuracy of screw placement in L5 is not very ideal. Indeed, L5 is the segment with the highest lumbar screw placement failure rate with free-hand screw placement or X-ray navigation only [12]. The pedicle morphology of the L5 vertebral body varies significantly at different stages of physical development [13]. Moreover, L5 vertebral body exhibit the largest standard deviation of both vertebral width and pedicle parameters [14]. It is widely believed that the rate of screw placement failure is significantly increased if the spinal canal at L5 exhibits a “clover” shape. The “clover leaf” spinal canal remains poorly understood, warranting further studies.

In this study, the risk factors of screw placement failure were analyzed by reviewing patients who underwent L5 pedicle screw fixation in our center over the past 12 months, measuring the L5 vertebral body parameters, and analyzing the corresponding screw placement accuracy rates.

## Materials and methods

We retrospectively analyzed patients in our spine surgery department who underwent L5 vertebrae fixation from 2020.10 to 2021.10. Patients who underwent lumbar multi-slice spiral computed tomography (CT) before and after surgery for lumbar disc herniation and lumbar spinal stenosis were included. To reduce the confounding effect of abnormal vertebral structure or rotation on screw placement, our exclusion criteria included 1. Lumbar spondylolisthesis; 2. Spondylolysis of L5 vertebral body; 3. Scoliosis (upper endplate L4–upper endplate S1 cobb Angle > 10°; or L5 upper endplate with horizontal angle > 5°); 4. L5 vertebral rotation (Nash-Moe Grade greater than or equal to grade 2 [15]); 5. Lumbosacral transitional vertebra. 6. Severe facet joint arthritis (Fujiwara Grade greater than or equal to grade 3 [16]).

A dual-source spiral 64-slice CT scanner (General Electric Company, USA) was used with the patients in the supine position.

During the analysis, we found that an increased screw placement failure rate paralleled a decrease in MCD. Therefore, we divided the patients into four groups, according to the mean and standard deviation of the MCD, A (MCD < 6 mm), B (6 mm < MCD < 8 mm), C (8 mm < MAC < 10 mm), and D (MCD > 10 mm) groups for measurement and analysis.

The Amiot grading system was used to evaluate the accuracy of pedicle screw placement, including levels 0–4: ideal position, 0–2 mm, 2–4 mm, 4–6 mm, and > 6 mm [17].

**Fig. 1:**
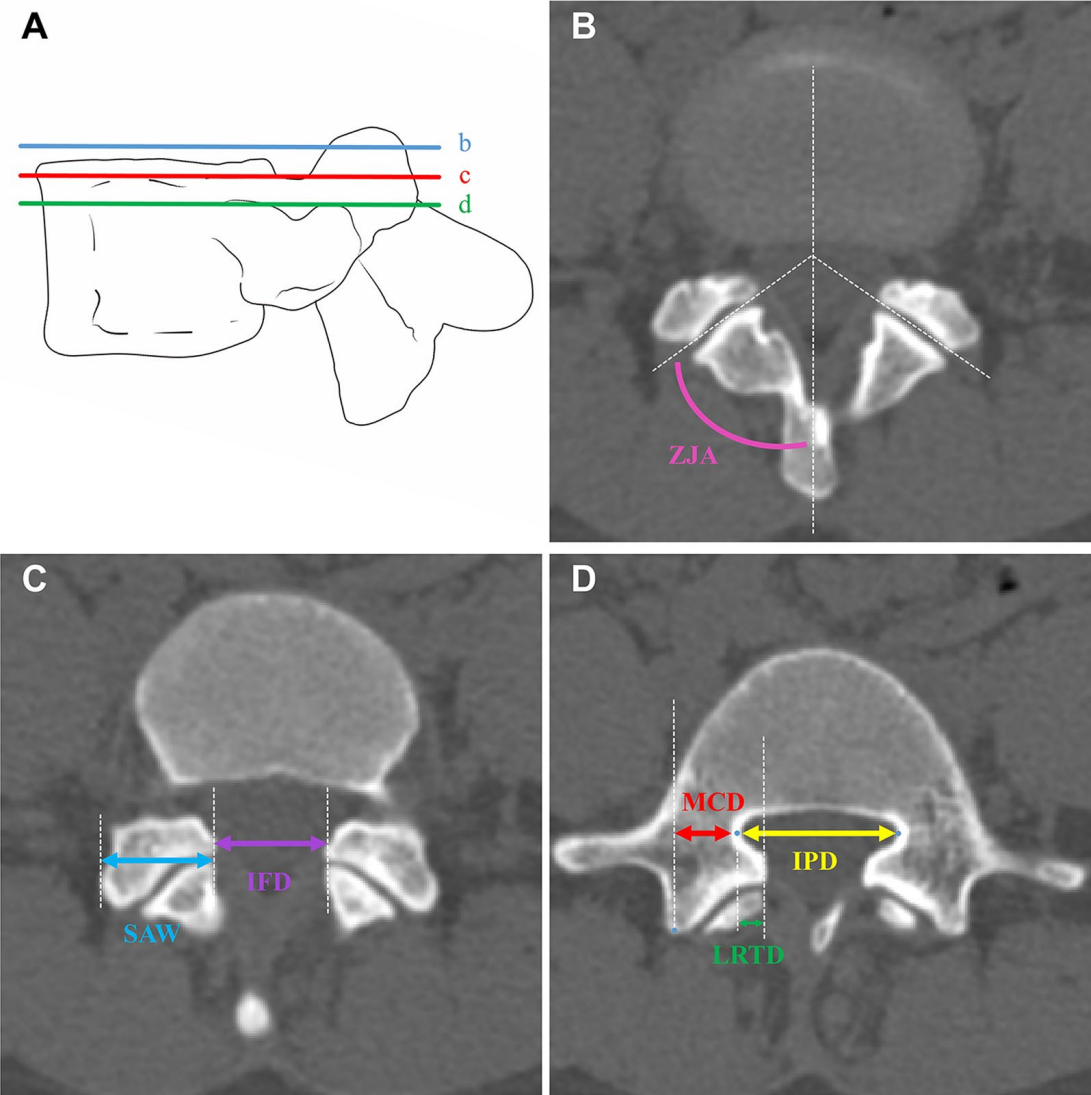
**A** Three axial sections parallel to the upper endplate: b. Through the midline of the disc; c. through the upper edge of the pedicle; d. through the medial margin of the pedicle. **B** ZJA was measured by the angle between the spinous process axis and the medial edge of the bilateral superior articular process. **C** IFD was measured by the distance between the inner edge of the bilateral superior articular process; SAW was measured by the length between the inner and outer edges of the superior articular process. D. MCD was measured by the vertical distance between the mammillary process and the inner edge of lateral pedicle; IPD was measured by the distance between the inner edges of bilateral pedicles; LRTD was measured by the distance between the superior articular process and the inner edge of the pedicle

Three reference lines parallel to the upper endplate were drawn from the axial CT scans across the midline of the upper disc, across the superior margin of the pedicle, and across the inner edge of the pedicle. Measurement of the ZJA in plane b was performed in accordance with a previously published study by Boden et al. [18]. The SAW and IFD were measured in plane c, and the MCD and IPD were measured in plane d. Meanwhile, the LRTD was measured by the horizontal distance from the medial edge of the superior articular process to the medial edge of the corresponding pedicle (Fig. 1).

**Fig. 2:**
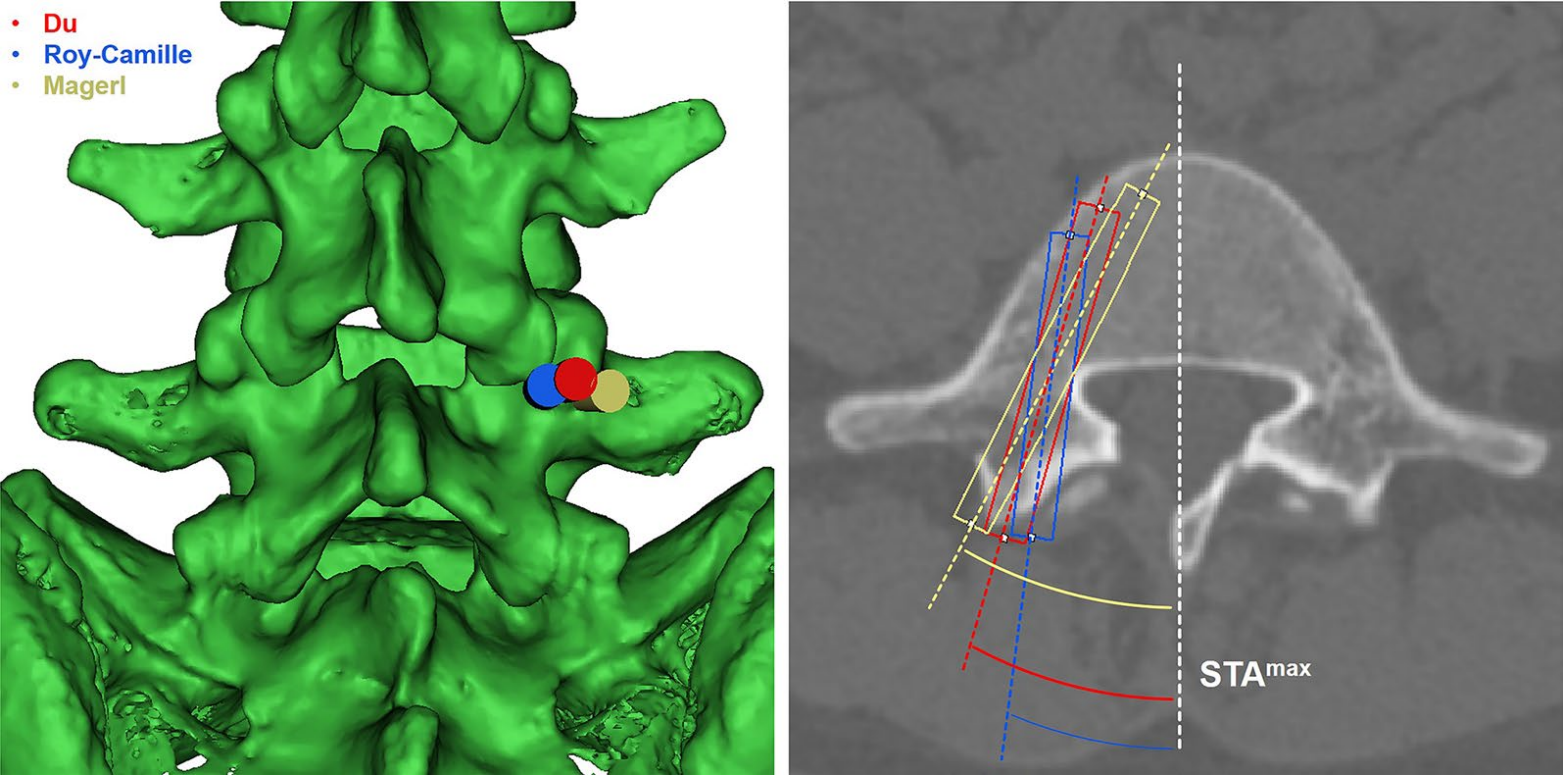
STA^max^ was measured by the angle of the long axis of the cylinder and the long axis of the spinous process when the simulated cylinder was tangent to the inner edge of the pedicle at different insertion points

The CT images were converted into 3D images of L5 vertebral bodies by Materialize interactive medical image control system version 21.0 (Mimics, Leuven, Flanders, Belgium). A 6.5-mm-diameter cylinder was used to simulate pedicle screws. The Magerl, Roy-Camille, and “^”-shaped crest techniques were used for the entry points, and the simulated screws were tangent to the medial pedicle wall. The STA^max^ was the angle between the pedicle screw and the line through the spinous process perpendicular to the posterior edge of the vertebral body (Fig. 2).

All statistical analyses were performed using SPSS 21.0 statistical software (SPSS, Chicago, IL, USA). An independent t-test was used to determine differences in pedicle parameters after stratifying by gender (male or female) and laterality (left or right). Analysis of variance (ANOVA) was used to examine the significance of differences in all pedicle parameters and demographics (age, height, weight, and BMI) between the A-D groups. The minimum significant difference was used for all comparisons between groups. Pearson correlation was used to examine the relationship between pedicle parameters and MCD. The chi-square test was used to compare categorical variables. A *P*-value < 0.05 was statistically significant.

## Results

A total of 299 patients (141 men and 158 women) with a mean age of 57.9 ± 12.87 years (32–83 years) underwent L5 vertebra fixation with 556 pedicle screws from October 20, 2020, to October 20, 2021 (Table 1). The pedicle screws were classified as grade 0 (*n* = 469, 84.4%), grade 1 (*n* = 38, 6.8%), grade 2 (*n* = 32, 5.7%), grade 3 (*n* = 5, 0.9%), and grade 4 (*n* = 12, 2.2%). The failure rates of screw placement in groups A to D were 65.7%, 9.0%, 11.2% and 2.2%, respectively (Table 2).

**Table 1:** Demographic date among groups

	Male	Female	Age	Height (cm)	Weight (kg)	BMI (kg/m^2^)
Group A	10	26	60.1 ± 11.1	157.4 ± 6.2	56.7 ± 7.9	22.6 ± 1.9
Group B	46	64	57.4 ± 11.2	158.3 ± 7.1	60.0 ± 6.9	24.0 ± 2.1
Group C	52	55	58.1 ± 13.5	160.0 ± 8.3	61.6 ± 7.4	24.2 ± 2.3
Group D	33	13	62.1 ± 13.2	165.0 ± 8.1	69.7 ± 6.4	25.5 ± 1.7
*P* value	0.324	0.146	0.031	< 0.001	0.016

**Table 2:** Accuracy of L5 screws placement

	Idea position	0.1–2.0 mm	2.1–4.0 mm	4.1–6.0 mm	> 6.0 mm	Totally
Group A	23 (34.3%)	18	18	3	5	67
Group B	189 (90.7%)	9	7	1	2	208
Group C	175 (88.9%)	10	6	1	5	197
Group D	82 (97.6%)	1	1	0	0	84

Parameters in group A (with minimum MCD) and group D (with maximum MCD) were significantly different from other groups. The smallest ZJA and IFD were observed in group A (17.8 ± 1.4 mm and 33° ± 5.9°, respectively). The largest bilateral LRTD (6.6 ± 1.6 and 6.3 ± 1.4) and smallest SAW (14.8 ± 1.5 mm and 14.4 ± 1.3 mm) were also found in group A. The l-SAW was significant difference between group A and other groups, with not a significant difference on r-SAW (Table 3).

**Table 3:** Difference of L5 pedicle parameters in group

	MCD (mm)	IPD (mm)	IFD (mm)	L-ZJA	R-ZJA	L-SAW (mm)	R-SAW (mm)	L-LRTD (mm)	R-LRTD (mm)
Group A	4.6 ± 1.2	28.3 ± 2.2	17.8 ± 1.4^*^	34.5 ± 6.4^*^	32.8 ± 5.9^*^	14.8 ± 1.5	14.4 ± 1.3*	6.6 ± 1.6^*^	6.3 ± 1.4^*^
Group B	7.0 ± 0.8	27.1 ± 2.7	21.0 ± 3.1	44.2 ± 7.4	45.0 ± 8.1	16.0 ± 1.7	15.5 ± 1.2	4.7 ± 1.4	4.3 ± 1.3
Group C	8.6 ± 0.7	26.7 ± 2.1	21.5 ± 2.9	47.6 ± 7.5	47.2 ± 7.9	15.5 ± 1.3	15.5 ± 1.1	3.8 ± 1.0	3.4 ± 1.3
Group D	11.2 ± 1.7	28.0 ± 1.4	24.3 ± 3.2^*^	47.3 ± 7.0	47.0 ± 7.0	16.1 ± 1.5	16.4 ± 1.3^*^	3.8 ± 1.1	2.9 ± 0.9
*P* value	< 0.001	0.549	< 0.001	< 0.001	< 0.001	0.071	0.034	< 0.001	< 0.001

*MCD* mammillary process—vertebral canal distance, *IPD* inter-pedicular diameter, *IFD* inter-facet distance, *ZJA* zygapophysial joint angle, *SAW* superior articular process width, *LRTD* lateral recess transverse diameter

*Compared with the other groups, the difference was statistically significant (*P* < 0.05)

**Fig. 3:**
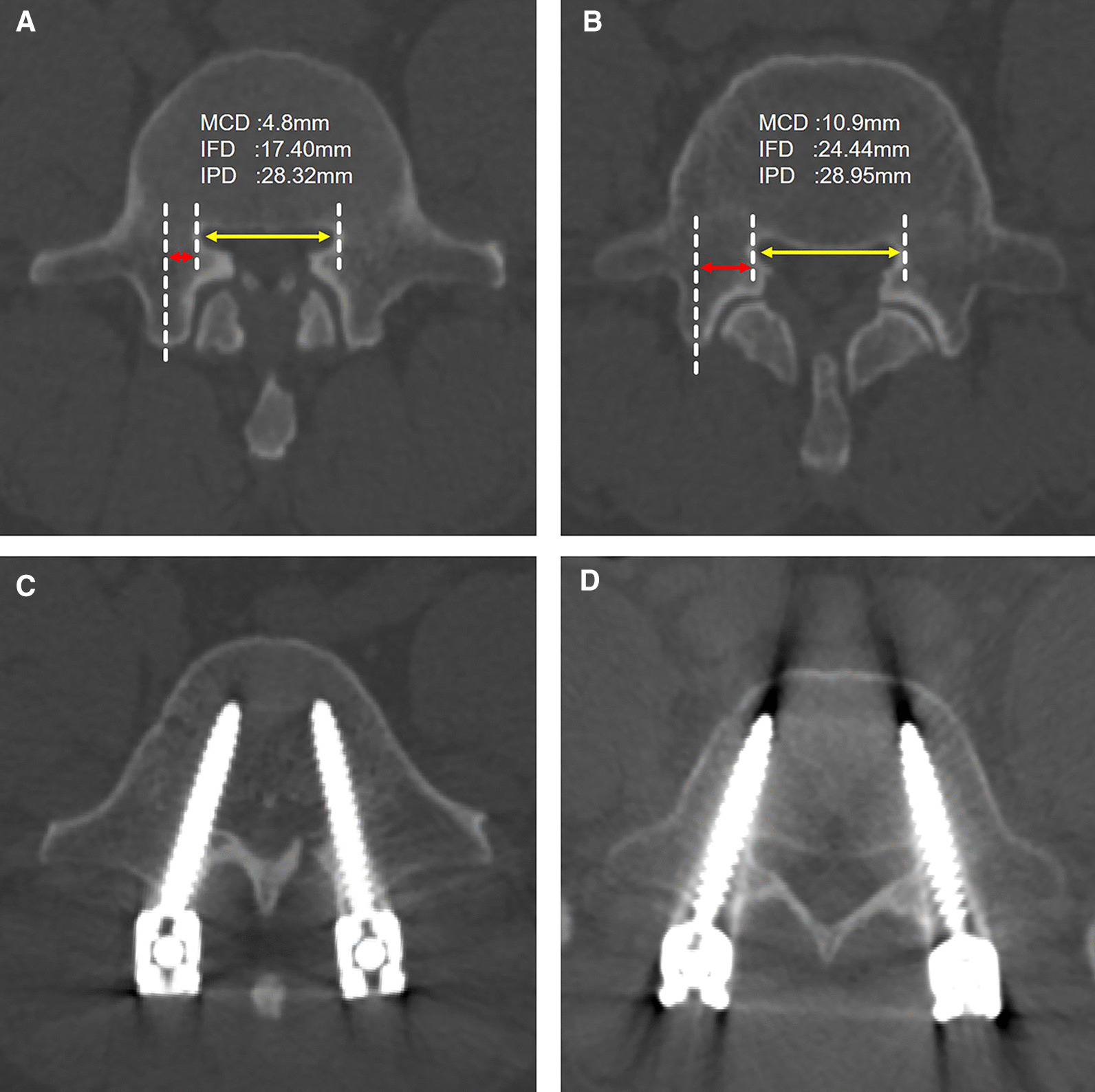
Screw placement was performed on L5 vertebrae with different MCDs. **A** Vertebral MCD < 6 mm; **B** Vertebral MCD > 6 mm; **C** Pedicle screw perforation through the pedicles (bilateral perforation > 4 mm); **D** Pedicle screws did not break through the pedicles

There was no significant correlation between MCD and IPD, while a positive correlation was found between MCD and IFD. Pearson’s correlation analysis showed that IFD positively correlated with MCD (*r* = 0.6976, *P* = 0.0001) (Fig. 3).

A smaller MCD was predominantly found in women. The proportion of female patients in group A was 72.2%. Further measurements showed that not only SAW was smaller than men in group A, but IFD was also smaller than men (Table 4).

**Table 4:** Difference of L5 pedicle parameters in sex

	Sex	MCD (mm)	IPD (mm)	IFD (mm)	L-ZJA	R-ZJA	L-SAW (mm)	R-SAW (mm)	L-LRTD (mm)	R-LRTD (mm)
Group A	F (26)	4.7 ± 1.0	27.2 ± 2.6	17.5 ± 1.4*	34.7 ± 8.2	33.0 ± 7.9	14.9 ± 1.4*	14.1 ± 1.5*	6.2 ± 1.4	6.2 ± 1.6
	M (10)	4.4 ± 0.9	28.8 ± 2.7	18.1 ± 0.9	34.0 ± 6.9	32.1 ± 5.1	16.2 ± 0.9	15.2 ± 1.3	6.3 ± 1.2	6.1 ± 1.7
Group B	F (64)	7.2 ± 0.4	27.1 ± 2.2	21.3 ± 1.1	44.8 ± 7.3	44.8 ± 8.1	16.2 ± 1.1	15.1 ± 2.3	5.0 ± 0.9	4.4 ± 1.1
	M (46)	6.8 ± 0.5	27.6 ± 2.5	22.3 ± 1.3	43.5 ± 8.0	45.2 ± 9.0	15.8 ± 1.2	16.1 ± 1.0	4.3 ± 1.1	4.0 ± 0.9
Group C	F (55)	8.8 ± 0.5	26.7 ± 3.1	21.6 ± 1.3	47.3 ± 8.3	46.4 ± 7.7	15.6 ± 1.1	16.0 ± 1.3	3.7 ± 1.0	3.6 ± 1.3
	M (52)	8.5 ± 1.4	26.7 ± 2.8	21.5 ± 2.0	47.9 ± 6.9	48.3 ± 7.6	15.3 ± 0.9	15.1 ± 1.3	3.0 ± 0.9	3.1 ± 1.1
Group D	F (13)	11.1 ± 1.9	27.6 ± 2.8	25.1 ± 1.8*	48.0 ± 7.1	48.2 ± 8.9	16.1 ± 1.0	16.5 ± 0.9	3.7 ± 0.8	2.6 ± 0.9
	M (33)	11.4 ± 1.7	28.4 ± 3.7	23.4 ± 1.5	46.8 ± 7.1	46.2 ± 7.1	16.2 ± 1.1	16.4 ± 1.5	4.0 ± 1.1	3.3 ± 1.0

*MCD* mammillary process—vertebral canal distance, *IFD* inter-facet distance, *IPD* inter-pedicular diameter, *ZJA* zygapophysial joint angle, *SAW* superior articular process width, *LRTD* lateral recess transverse diameter

*Compared with the male groups, the difference was statistically significant (*P* < 0.05)

As MCD decreases, STA^max^ decreases. The STA^max^ of group A was only 3.68° by the Magerl technique. The STA^max^ was about 15–16° for groups B and C and 25°for group D (Table 5).

**Table 5:** STA^max^ in different entry points

	Group A	Group B	Group C	Group D
Magerl	3.68 ± 3.90	14.96 ± 3.63	15.96 ± 4.64	24.23 ± 3.63
Roy-Camille	− 8.17 ± 3.17	2.74 ± 2.56	4.67 ± 2.11	10.78 ± 4.1
Du	− 1.12 ± 2.96	9.98 ± 2.42	12.18 ± 3.01	17.54 ± 2.92

*STA*^*max*^ maximum safe transverse angle

**Fig. 4:**
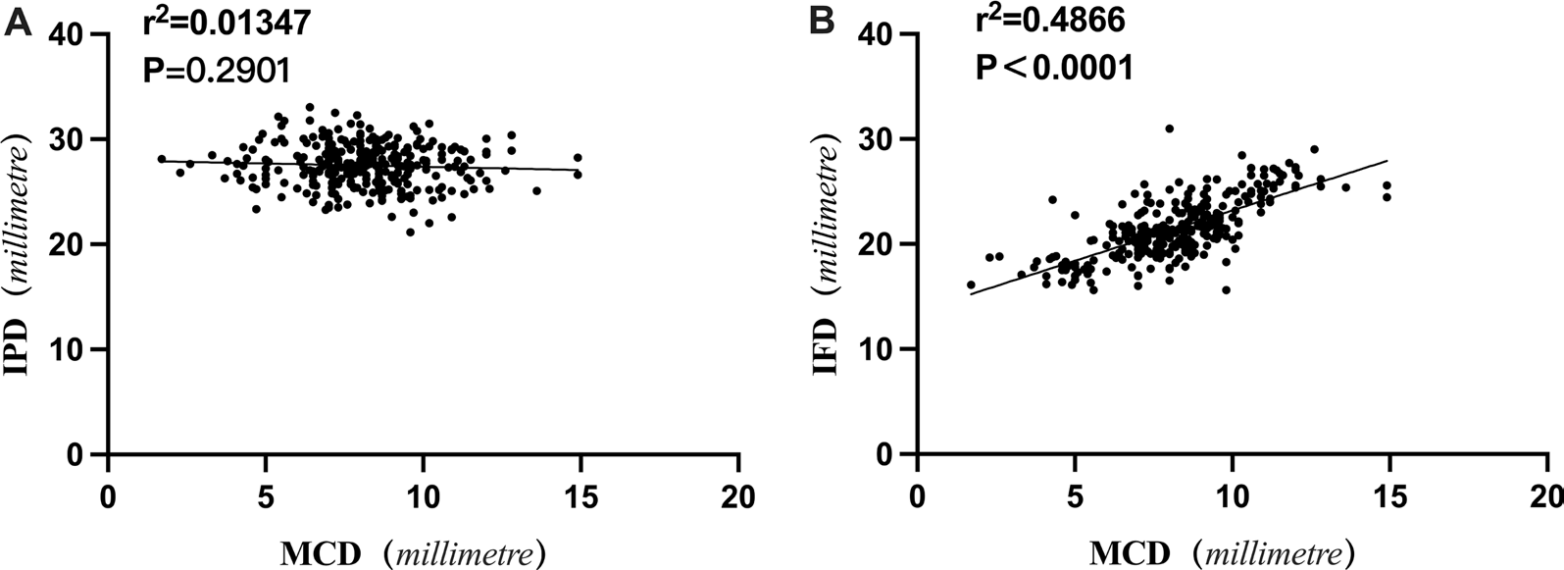
Relationship between L5 parameters and MCD. **A** IPD was not significantly correlated with MCD. **B** IPD was significantly correlated with MCD

Interestingly, during the measurement process, we found a simple preoperative evaluation method. In preoperative horizontal or vertical CT cross sections, when a vertical line passing through the mammillary process was medial to the midpoint of the pedicle, it indicated that the superior articular process was too close to each other. This phenomenon was found in 93% of cases in Group A and 5% in the other groups (Fig. 4).

## Discussions

Navigation technology has developed rapidly in recent years. Current evidence suggests that the accuracy of robotic screws placement or intraoperative CT navigation is higher than free-hand or intraoperative X-ray navigation [19]. However, this navigation equipment is expensive, and most spinal surgeons still place pedicle screws free-hand or with intraoperative X-ray. The accuracy of free-hand screw placement is not ideal, especially for L5. An increasing body of evidence suggests that during placement of L5 segment, it should be directed outwards and upwards [5, 20]. Measurement and analysis of CT images suggest that the entry points for L5 have a tendency to be inward, requiring either the “^”-shaped crest or Magerl technique.

**Fig. 5:**
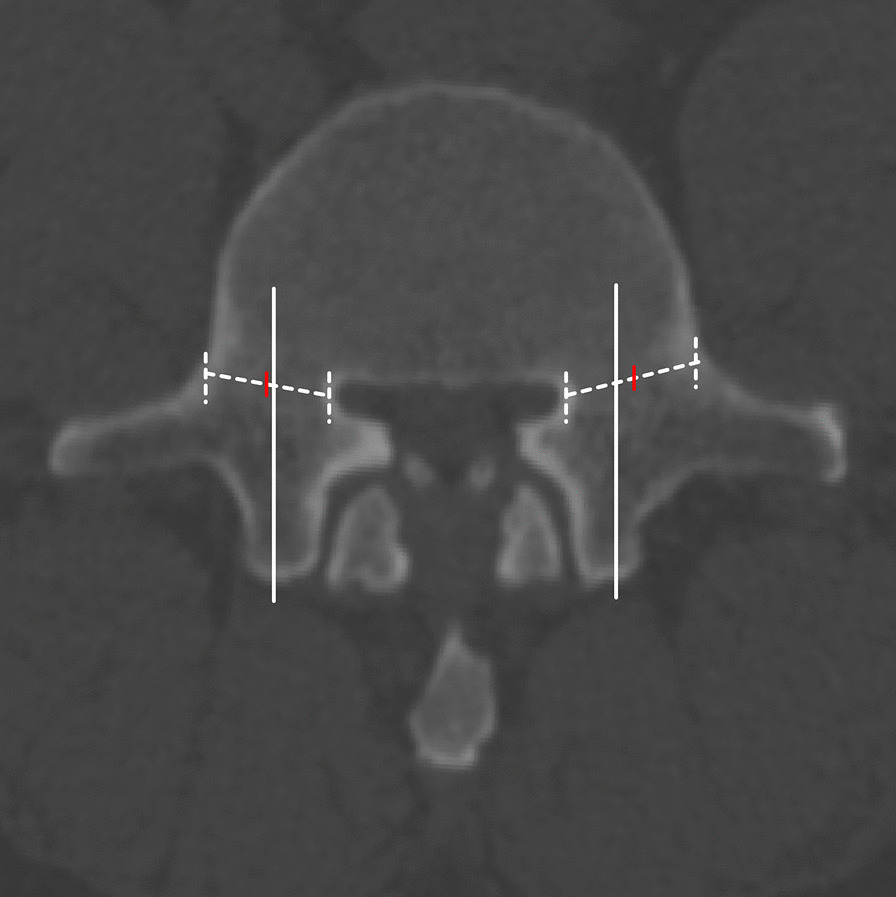
The vertical line through the mammillary process is medial to the midpoint of the pedicle

At present, the commonly used screw placement entry points use the superior articular process and transverse process as the reference. However, the transverse process of the lower lumbar spine is deep and sometimes blocked by the iliac crest and pulled by the erector spine muscle; accordingly, it is difficult to fully expose the root of the transverse process during surgery. Therefore, the morphology of the superior articular process and its position relative to the spinal canal directly affect the accuracy of pedicle screw placement. When the superior articular process faces inward, the insertion point is also inward relative to the spinal canal. In this case, screw placement at an angle of 10–15° can cause the screw to penetrate the inner wall of the pedicle (Fig. 5). Importantly, we found that when the MCD was less than 6 mm, the screw placement failure rate increased significantly. Evaluation of the preoperative CT showed that the STA^max^ of screw placement was only 1–7°. For these patients, the angle of insertion should be abducted if the screws are placed at commonly used entry points.

In fact, such cases are not uncommon. More than 10% of the 299 patients (*n* = 36) presented with a low MCD, which may be attributed to selection bias caused by the smaller ZJA [21]^.^

All 299 patients included in this study were patients with lumbar disc herniation or lumbar spinal stenosis. Although patients with severe facet arthritis were excluded at the time of inclusion, changes in facet joint morphology could affect the final measurements [22, 23]. The ZJA at L5 is generally about 45° and does not exhibit significant heterogeneity across different populations. The reported average ZJA in Indian [24], Chinese [25], and French [26] populations is 48.32°, 47.7°, and 44.4°, respectively. In the present study, the average ZJA of L5 (*n* = 598) was 46.3°, consistent with the literature. However, the ZJA in group A decreased significantly to 33.7°. Morphologically, this type of facet joint is more similar to the superior facet of L4. When the L5 superior facet became more sagittal than horizontal, it increased susceptibility to degenerative lumbar diseases, including lumbar disc herniation and degenerative lumbar spondylolisthesis [22, 27]. Excessively small vertebral bodies and steep facet joints were associated with lumbar instability, while excessively steep facet joints could accelerate the rate of disc degeneration [28].

**Fig. 6:**
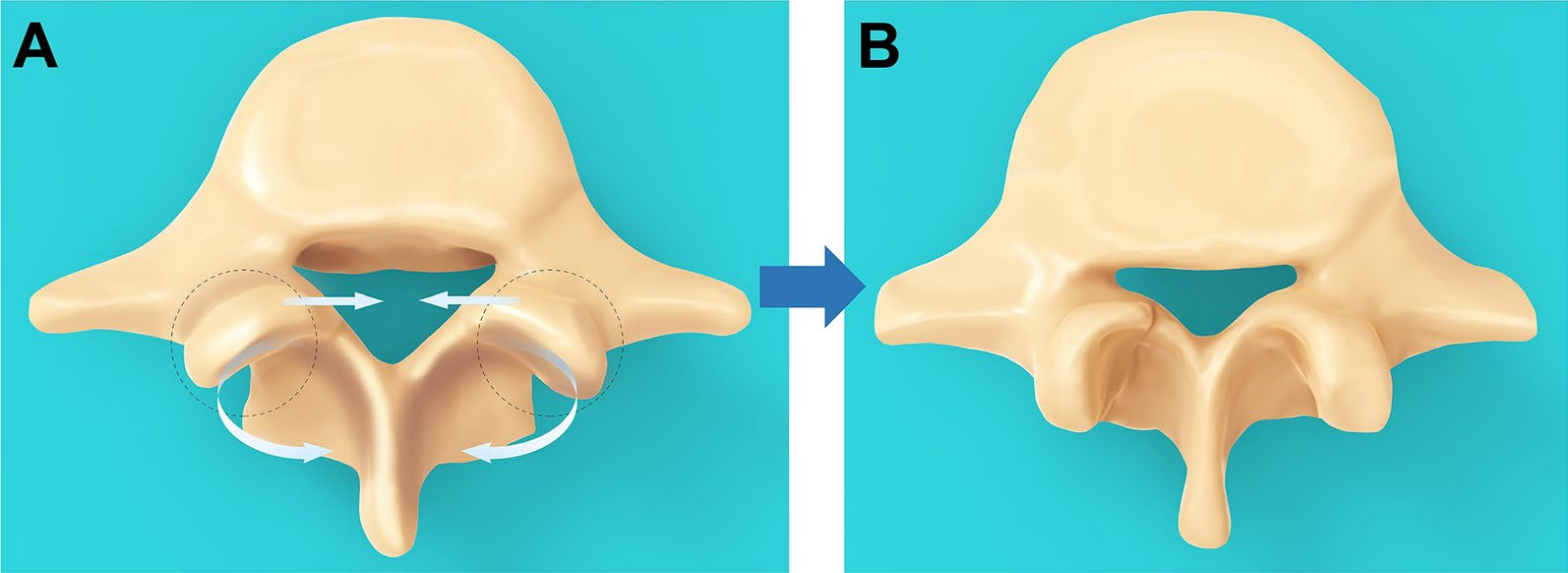
Illustration demonstrating the difference between the two types of superior articular processes. In groups with low MCD (**B**), the superior articular processes are closer to each other and rotate inward compared to groups with normal MCD (**A**). At this point, the spinal canal exhibits a "clover" shape

Moreover, we found that the small distance between superior articular processes of L5 was associated with a small MCD. A study by Santiago et al. where the sagittal diameters of lumbar vertebrae and articular processes of 39 healthy Spaniards were measured showed the distance between L5 superior articular processes was 22.2 mm ± 0.40 mm [29]. Consistently, Oguz et al. measured the lumbar vertebrae of 24 healthy Turks, and the distance between L5 superior articular processes was 21.7 mm ± 2.8 mm [30]. In this study, the inter-facet distance in group A was only 17.8 mm ± 1.4 mm, indicating that the superior articular processes of group A are closer to each other than in other groups. In addition, the SAW was also smaller in group A compared with BCD, although this reduction was not statistically significant on one side. Compared with the normal superior articular process, the superior joint of group A exhibited a steeper and sharper variation. The steep superior articular process tended to narrow the spinal canal compared to the gentle superior articular process. The L5 spinal canal of this type of superior articular process typically exhibits a “clover” shape, with the superior articular process extending inward (Fig. 6).

IPD is also a factor that affects the relative position of the entry point. However, the IPD in group A was only 28.3 ± 2.2 mm and did not increase significantly. Meanwhile, the overall mean IPD was 28.4 mm ± 2.3 mm, which was not significantly different from the reported average transverse diameter in Chinese (29.61 mm ± 0.63 mm) and Indian (28.02 mm ± 0.37 mm) populations in the literature [31].

Mitra, Datir, and Hou et al. observed a sequential outward migration of the central entry point of the lumbar pedicle from L1–L4 [14, 32]. At L5, the entry point is located lateral to the lateral boundary of the facet joint. However, if the angle of the superior facet of L5 is small with no “abduction,” the usual Magerl entry point is inward-facing. In some medical centers, to avoid this kind of situation, the root of the transverse process is adopted for screw insertion, which is more lateral than Margel entry point, with an accuracy rate of > 98% [33]. However, a more external entry point means more muscle dissection, bleeding, and longer surgical time. Although screw placement failure was avoided in a small number of patients, the cost-effectiveness of this approach needs further study. Based on the current Magerl or “^”-shaped crest insertion methods, preoperative estimation of excessive orientation of the L5 superior articular process is a cost-effective method. When the MCD is less than 6 mm, the internal angle of screw placement should be reduced, and vertical entry into the vertebral body may be required.

## Conclusions

We report a distinct superior articular process of the L5 vertebral body characterized by MCD < 6 mm, decreased IFD, SAW, and ZJA. In this type of vertebral body, the superior articular process is closer to the spinal canal when used as the reference for the traditional insertion point, increasing the risk of spinal canal invasion during pedicle screw placement. Preoperative evaluation of the superior articular process on CT images is vital to avoid screw perforation into the spinal canal.
